# Graft healing in anterior cruciate ligament reconstruction

**DOI:** 10.1186/1758-2555-1-21

**Published:** 2009-09-23

**Authors:** Chih-Hwa Chen

**Affiliations:** 1 Department of Orthopaedic Surgery, Chang Gung Memorial Hospital at Keelung, Chang Gung University College of Medicine, Taoyuan, Taiwan

## Abstract

Successful anterior cruciate ligament reconstruction with a tendon graft necessitates solid healing of the tendon graft in the bone tunnel. Improvement of graft healing to bone is crucial for facilitating an early and aggressive rehabilitation and ensuring rapid return to pre-injury levels activity. Tendon graft healing in a bone tunnel requires bone ingrowth into the tendon. Indirect Sharpey fiber formation and direct fibrocartilage fixation confer different anchorage strength and interface properties at the tendon-bone interface. For enhancing tendon graft-to-bone healing, we introduce a strategy that includes the use of periosteum, hydrogel supplemented with periosteal progenitor cells and bone morphogenetic protein-2, and a periosteal progenitor cell sheet. Future studies include the use of cytokines, gene therapy, stem cells, platelet-rich plasma, and mechanical stress for tendon-to-bone healing. These strategies are currently under investigation, and will be applied in the clinical setting in the near future.

## Graft healing

One of the most challenging and important problems physicians are facing is the failure of anterior cruciate ligament (ACL) reconstruction after injury and primary surgical repair [1-4]. It is possible to ensure a good clinical outcome of the primary repair in carefully selected patients [5]. The cause of failure of the ACL has been debated. It has been suggested that the behavior of cells differs, suggesting that uniquely disabled fibroblasts populate the ACL. The unsatisfactory healing is due to the failure of the cells and blood vessels within and around the ACL to mount an adequate healing response, to bridge the gap between the ruptured ends of the ACL, and the lack of the wound-site to fill within the intra-articular environment [6-9].

ACL reconstruction using semitendinosus and gracilis tendons has become popular in recent years. Tendon-to-bone incorporation of the tendon graft within a bone tunnel is a main concern when using a tendon graft for ligament reconstruction. Successful ACL reconstruction with a tendon graft requires solid healing of the tendon graft in the bone tunnel as soon as possible after surgery. Enhancing the healing of the tendon graft to the bone is crucial to facilitate an early and aggressive rehabilitation and a rapid return to full activity. The basic biology of the tendon graft-bone tunnel healing remains incompletely understood. Distinct variability occurred in the morphological characteristics of the healing at the site of attachment of the tendon and the bone. The tendon-to-bone healing in a bone tunnel occurs by bone ingrowth into the fibrovascular interface tissue that initially forms between the tendon and the bone. First, progressive mineralization of the interface tissue occurs. Subsequently, the bone grows into the outer tendon and the tendon graft becomes incorporated into the surrounding bone. Progressive re-establishment of the continuity of collagen fibers between the tendon and the bone results in restoration of a tendon-bone junction [10-16].

The primary site of weakness during the early postoperative period is the tendon-bone interface, particularly while the tendon attaches to the bone within the intraarticular environment. The osteointegration of the tendon grafts used for the replacement of an ACL may still be unsatisfactory and may be associated with postoperative anterior-posterior laxity. The firm attachment of the tendon graft to the bone allows earlier and more aggressive rehabilitation and a quicker return to full activity.

To improve the healing of the ACL graft, new strategies to promote the intra-articular and intraosseous healing are evolving. Although these strategies are currently under investigation, they are expected to be clinically applied in the near future. The strategies to improve the tendon-to-bone tunnel healing focus on providing appropriate molecular signaling thereby allowing cell differentiation which result in an effective healing response between the tendon and the bone. A sufficient population of stem cells is required for optimal tissue regeneration. The mesenchymal stem cell-treated grafts have cartilage at the tendon-bone interface [17-20]. Bone ingrowth plays an important role in the graft-to-bone fixation. Several strategies have been demonstrated to improve bone ingrowth into a tendon graft that was placed in a bone tunnel. Most of these strategies involved the use of osteoinductive cytokines [21-25]. The tendon graft-to-bone healing could be improved by the use of many factors, such as brushite calcium phosphate cement, injectable tricalcium phosphate, mesenchymal stem cells, hyperbaric oxygen treatment, transforming growth factor-beta 1, calcium-phosphate, bone marrow, demineralized bone matrix, synovial mesenchymal stem cells, granulocyte colony-stimulating factor, magnesium-based bone adhesive, bone morphogenetic proteins-2 (BMP-2), low-intensity pulsed ultrasound and shock wave therapy [17-32]. Osteoconductive materials may also play a role in improving the healing of the tendon in the bone tunnel via enriched bone ingrowth [26,27,29-31]. Shock wave treatment significantly improves the healing rate of the tendon-bone interface resulting in significantly more trabecular bone around the tendons [26]. Hyperbaric oxygen treatment, low-intensity pulsed ultrasound and extracorporeal shockwave could induce a marked increase in vascularity that improves the formation of new bone [28,31,32].

These various methods demonstrate the challenge of achieving a secure biologic fixation of the tendon graft in a bone tunnel with the current ACL reconstruction techniques.

Our strategy to enhance the tendon-to-bone healing is to use the periosteum, hydrogel with periosteal progenitor cells (PPCs) and BMP-2 and PPC sheets.

## Periosteum to enhance tendon-to-bone healing

The periosteum consists of multipotent mesodermal cells. In addition, it contains chondroprogenitor and osteoprogenitor cells, which can form both cartilage and bone under appropriate conditions [33-37]. The periosteal tissue may be used to improve the healing between the tendon graft and the bone. In our experimental studies, we evaluated the effect of periosteum-enveloping tendon graft on tendon-to-bone healing in two different experimental models in rabbits: periosteum-enveloping tendon graft in a bone tunnel, and periosteum-enveloping tendon graft in ACL reconstruction [38,39].

In the bone tunnel model, cross sections of the bone tunnel showed that the periosteal tissue formed a fibrovascular interface between the tendon and the bone. Due to the new bone formation, the cancellous bone in the bone tunnel interdigitated with the fibrous interface tissue four weeks after operation. There was progressive mineralization and maturation of the new bone that grew into the interface fibrous layer. There appeared to be excellent integration between the fibrous interface layer and the bone and between the tendon and the interface layer at eight weeks after operation. Progressive collagen fiber-bone anchoring, maturation and organization between the tendon and the bone lining cells occurred. In addition, fibrocartilage formed between the tendon and the bone [38].

In the ACL reconstruction model, radiographs showed bone resorption as well as the formation of new bone around the femoral and tibial bone tunnel in the periosteum-treated tendon graft. There was further matrix deposition at the tendon-to-bone interface. Eight weeks after operation, the periosteum-enveloping specimens demonstrated cartilage and bone formation around the tendon graft in the femoral and tibial tunnel. There was extensive formation of new bone trabeculae and cartilage at the tendon-to-bone interface with new bone direct apposition to the tendon [39].

**Figure 1:**
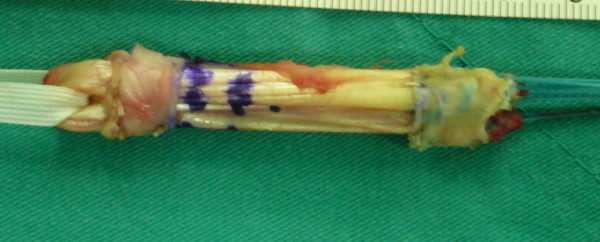
**ACL reconstruction with periosteum-enveloping hamstring tendon autograft**.

**Figure 2:**
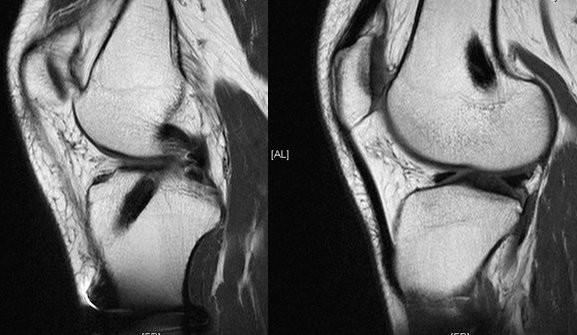
**MR image of the tendon graft in the bone tunnel showed excellent incorporation**.

The periosteum-treated tendon graft was used for ACL reconstruction to enhance the healing of the tendon graft in the bone tunnel. The graft was composed of double loops of semitendinosus and gracilis tendons. A periosteal flap was harvested from the anterior tibial cortex and divided into two flaps. The periosteum was wrapped with the cambium layer, placed outside to face the tunnel wall and then sutured on the tendon at both sides where the tendon graft approaches the tunnel opening [40](Fig. 1). In the follow-up study, clinical assessments with the Lysholm knee score showed progression of 59 to 94 points before and after surgery. After reconstruction, 81% of the patients could return to moderate or strenuous activity. 94% of the patients were assessed as normal or nearly normal according to the International Knee Documentation Committee (IKDC) guideline. A bone tunnel enlargement of more than 1 mm was identified in 5% of the femoral tunnel and 6% of the tibial tunnel [41]. The periosteum can be easily harvested from the proximal tibia by routine incision to harvest hamstring tendons. In addition to its potential in improving the tendon-to-bone healing, the enveloped-periosteum may help to seal the intra-articular tunnel opening quickly after surgery, thus avoiding the reflux of synovial fluid into the tunnel. Subsequently, the bone tunnel enlargement could be reduced [35](Fig. 2).

## Hydrogel with PPCs and BMP-2 to enhance tendon graft-bone healing

PPCs have the potential to differentiate into osteogenitor and chondrogenitor cells in an adequate microenvironment. PPCs are used to enhance the tendon-to-bone healing process by forming a fibrocartilage interface. A novel injectable hydrogel supplemented with PPCs and BMP-2 was developed for an easier delivery into the bone tunnel.

Photopolymerization allows for an impressive degree of spatial and temporal control with implications for diverse, minimally invasive applications for tissue regeneration. [42-50] PPCs require appropriate signals to differentiate into cartilage and bone. BMP-induced signal transduction is an important positive regulator. PEGDA-based polymers (polyethyleneglycoldiacrylate) can provide a suitable microenvironment for the growth and differentiation of mesenchymal stem cells [51-55].

Hyaluronic acid (HA) is applied for biomaterial fabrication and application to transduce intrinsic signals within a structure and to enhance tissue formation. In addition, it plays a crucial role in promoting cell differentiation and cell growth [56,57]. In our experimental studies, the effect of a hydrogel supplemented with PPCs and BMP-2 on tendon-to-bone healing was evaluated in two animal models: tendon graft in a bone tunnel model and tendon graft in an ACL reconstruction model [58,59].

In the bone tunnel model, we tested the feasibility of HA tethered to BMP-2 to stimulate PPCs to direct the fibrocartilagenous attachment and new bone formation in an extra-articular tendon-to-bone healing model. The PPC-BMP-2 hydrogel was injected and photogelated in the bone tunnel after placing the tendon graft into the bone tunnel. The histological analysis showed that interface fibrocartilage and new bone formed within six weeks. The healing of the tendon-to-bone interface underwent a gradual remodeling process. The biomechanical testing revealed a higher maximum pull-out strength and stiffness with a statistically significant difference. It appears that photoencapsulation of BMP-2 and PPCs has a powerful inductive ability for the healing between tendon and bone [58].

**Figure 3:**
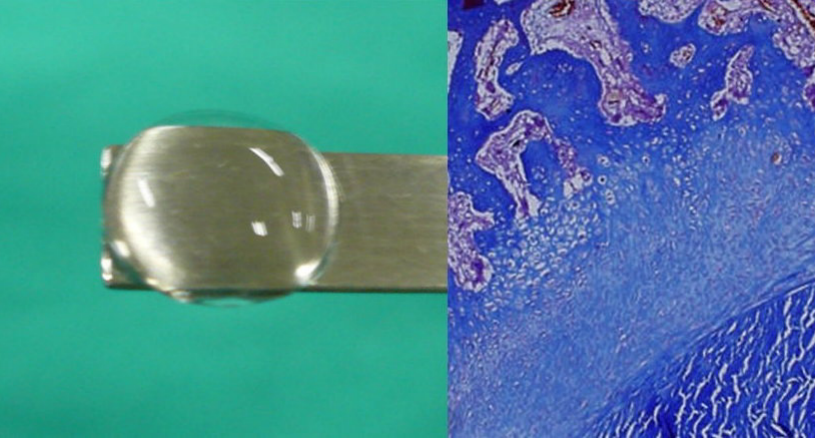
**Injectable hydrogel to enhance the tendon graft-bone healing**.

In the ACL reconstruction model, the PPC-BMP-2 hydrogel was injected and photogelated in the femoral and tibial tunnel after ACL reconstruction with a flexor digitorum longus tendon in rabbits. Histological analysis of the tendon-to-bone interface in the femoral and tibial tunnel showed that an interface layer was formed by the hydrogel. After eight weeks, there was further matrix deposition with fibrocartilage formation at the tendon-to-bone junction. After 12 weeks, large areas of fibrocartilage at the tendon-to-bone junction formed an interface. The use of the PEGDA-based hydrogel provided an adequate matrix for the encapsulation of cells and signal factors. In addition, it was an effective local delivery method to reach the bone tunnel through injection. After the hydrogel is injected, it can be solidified via a photoinitiated polymerization process which ensures encapsulation of the stem cells and growth factors [59] (Fig. 3).

## PPC sheet to enhance tendon-to-bone healing

**Figure 4:**
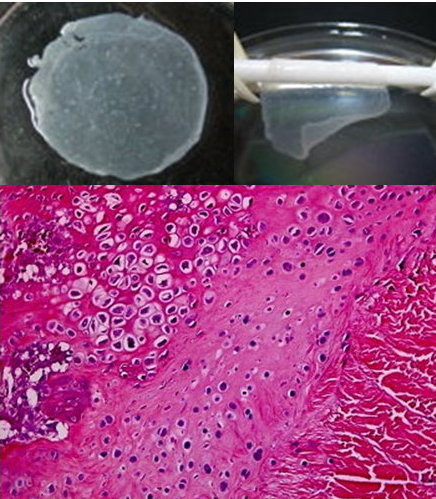
**Cell sheet act as artificial periosteum to ehhance the tendon-bone healing**.

A scaffold-free method using polymerized fibrin-coated dishes to generate functional PPC sheets that can be used as periosteum-like tissue transplants was developed. Polymerized fibrin-coated polyethylene dishes were fabricated with fibrinogen monomers which were mixed with thrombin. PPCs derived from the periosteum of the tibia of rabbits were cultivated on a fibrin-coated surface. The laminated cell sheets were detached from the polymerized fibrin layer by proteases secreted from the cells. The PPC sheets were seeded onto the small intestinal submucosa (SIS) layer. The PPC sheets could be harvested non-invasively as intact, transplantable sheets by using an intrinsic protease. The PPC sheets were wrapped around the tendon grafts which were then used for ACL reconstruction in rabbits. There was further matrix deposition with formation of fibrocartilage at the tendon-to-bone junction after eight weeks. The bioengineered PPC sheets and SIS co-layer cell sheet act as artificial periosteum which offers a novel therapeutic strategy to augment the healing at the tendon-to-bone junction (Fig. 4).

## Discussion

Current techniques of ACL graft reconstruction require healing of a tendon graft in a bone tunnel. The attachment site of the graft to the bone differs from the human anatomy where ligaments attach to the surface of bone. There are no sites in the body where a tendon is surrounded by bone such as in a bone tunnel. A common cause for an unsatisfactory ACL reconstruction is a failure of the graft-to-bone healing. Three fundamental factors may be responsible for the lack of cell signaling and differentiation resulting in an ineffective healing response between the tendon and the bone. These factors include the presence of inflammation in the postnatal organism, tendon-to-bone interface motion and an insufficient number of undifferentiated cells at the healing tendon-to-bone interface.

The successful ACL reconstruction with a tendon graft necessitates effective healing of the tendon graft in the femoral and tibial bone tunnel. The tendon-to-bone healing progresses via an interzone of vascular, highly cellular fibrous tissue, which undergoes a maturation process that lasts until its matrix consists of oriented collagen fibers and the fibrous interface becomes indistinctable. [10,60] The tendon-to-bone healing in a bone tunnel occurs via bone ingrowth into the fibrovascular interface tissue that forms between the tendon and the bone. Progressive re-establishment of collagen fiber continuity between the tendon and bone facilitates the formation of the tendoosseous junction. [2] The development of Sharpey-like collagen fibers that connect the tendon graft to the bone has been described and is viewed as the earliest sign of osteointegration [61].

When a bone-patellar tendon-bone (BPTB) graft is used for ACL reconstruction, the fixation of the graft depends on the bone-to-bone healing. However, the length of the patella tendon portion of most BPTB grafts is greater than the intra-articular length of the ACL, resulting in tendon-to-bone healing (between the tendon portion and the tibial tunnel) rather than bone-to-bone healing. It is believed that the graft fixation strength is lower and the healing is poorer in the tendon graft compared with the bone plug healing in the tunnel. In the tendon graft, the weakest site was the graft-wall interface at three weeks and the intraosseous tendon graft at six weeks [62].

Spatial and temporal differences in the tendon-to-bone healing exist in the different regions of the bone tunnel. The healing of the tendon-to-bone interface tissue shows a wide chondroid matrix at the intra-articular aperture, in contrast to a narrow, fibrous matrix at the intra-osseous portion. The collagen continuity between the tendon graft and the bone tunnel increased over time, with a more parallel orientation and increased collagen fiber continuity between the tendon and the bone. Significant differences in the healing between the tendon graft and the bone exist throughout the length of bone tunnel. The etiology of these differences includes a variable biological and biomechanical environment at different sites of the tunnel. [63] Adequate mechanical loading on the tendon graft should be one of the basic requirements for long-time survival of the graft tissue. The tendon healing in a bone tunnel is influenced by mechanical stress. It has been shown that the differentiation of mesenchymal stem cells is directly influenced by pressure and tension. In the bone tunnel, mechanical loading occurs mainly by shear forces, which might prevent or delay the development of a fibrocartilage zone and leads to the development of an indirect insertion [64].

On the basis of a normal ACL structure and the known function of the insertion site, the ideal tendon graft would attach broadly to the surface of the bone at the site of the femur and tibia by an intermediate zone of fibrocartilage.

The periosteum contains multipotent mesodermal cells. It has been shown that the environment has an influence on the differentiation of cells in free periosteal grafts [40]. The periosteum has osteogenic capacity and can promote the formation of cartilage in a chondrotrophic environment. The periosteum has also the ability to initiate the formation of endochondral bone by inducing the differentiation of mesenchymal cells into chondroblasts and subsequently into osteoblasts. In addition, the periosteum can also augment bone ingrowth into the collagenous tissue thereby inducing ossification and bone formation. Free autologous periosteal transplants have been reported to produce hyaline-like cartilage in chondral defects of the patella, which suggests the potential of stem cells in the cambium layer to produce cartilage [65]. Thus, based on the findings of these studies, if the periosteum is applied to the surface of a tendon graft it should form cartilage or bone tissue. In addition, if the periosteum-enveloping tendon graft is placed extra- or intraarticularly in a tunnel, it may promote the initiation and regulation of the bone ingrowth into the tendon graft.

The cambium layer serves as a fibrous layer between tendon and bone when the periosteum is sutured on the surface of the tendon and transplanted into a bone tunnel. The bone ingrowth into the cambium layer as well as the interdigitation between the periosteal tissue and tendon can be observed after four weeks. The interface of the fibrous layer, which originates from the wrapped periosteum, becomes progressively incorporated. The organization of the interface develops over time. There is an extensive formation of fibrocartilage at the tendon-bone interface. The periosteum has the powerful inductive ability to enhance the healing between the tendon and the bone tunnel. The periosteum can induce the differentiation of mononuclear cells into chondroblastic and osteoblastic cells. Subsequently, it directs the production of fibrocartilage or osteoid followed by mineralization and progressive remodeling which occurs during the healing process [38]. The periosteum has a rapid effect on bone ingrowth thereby enhancing the strength of the fixation. The enveloping of the tendon graft with the periosteum may be an effective way to avoid delayed graft healing. The periosteum may be even more effective in situations in which healing is impaired, e.g. in those with a widened bone tunnel in a revision operation.

The expansion of the tunnel is significantly greater following ACL reconstruction using hamstring tendon autografts. This is because of the greater distance between the normal insertion site and the biomechanical point of action of the ACL. The greater distance creates a potentially larger force moment during graft cycling which may lead to the greater expansion of the bone tunnel [66]. Adequate and earlier tendon-to-bone healing may solve this problem. This technique may be applied to ACL reconstruction to enhance the tendon graft healing within the tunnel.

In order to enhance the tendon-to-bone tunnel healing, our laboratory intended to develop an injectable hydrogel to fill the tendon-bone tunnel interface in a tissue-engineering approach. Tissue-engineering therapies for biomimetic material rely on the stimulation of signaling growth factors to induce cellular chemotaxis, proliferation, differentiation and a notable formation of new tissue at the required site. Recent studies have shown that various growth factors play important roles in tissue repair both in vivo and in vitro [26,61,67-72]. Therefore, photopolymerizable hydrogel supplemented with PPC and BMP-2 would also enhance the formation of an interpenetrating network that may limit the tunnel enlargement and improve the healing response of the graft in the tunnel. Photopolymerized hydrogel is used as scaffold material in tissue-engineering techniques and typically serves as carrier or filler in biological systems. Scaffolds should, in part, mimic the structure and biological function of an extracellular matrix. They should promote cell proliferation, induce cell differentiation or enhance the growth of surrounding tissues. Numerous biological molecules have been used as functional additives in hydrogel to enhance the development of engineered tissues. Biological molecules, such as growth factors, together with cells can be physically encapsulated in the hydrogel [57]. In addition, growth factors can be covalently tethered to the hydrogel to prevent their loss by leaching or extraction [73]. This tethering offers prolonged retention of those growth factors to induce the relevant signaling pathways. The addition of BMP-2 to the hydrogel for its delivery to the target site is essential to allow BMP-2 to act there. Photoencapsulation of BMP-2 in a PEGDA-based hydrogel provides the temporally and spatially defined modulation of chondroblastic and osteoblastic differentiation. Consistent stimulation by prolonged BMP-2 retention enhances the migration and proliferation of PPCs. However, several critical problems must be addressed before the clinical application of this technique, such as dose determination, a simple and reliable delivery, the maintaining of the effect in the tunnel and the cost effectiveness of this application.

Based on the finding that the tendon-to-bone healing progresses by bone ingrowth into the fibrous tissue interzone, exogenous osteoinductive agents should be used to augment this process. We demonstrated improved healing by applying injectable photopolymerizable hydrogel at the tendon-to-bone interface [44,74]. Our study suggests that photoencapsulation of BMP-2 and PPCs has the ability to improve the healing between the tendon and the bone. This technique may provide a novel platform for tissue-engineered stem cell therapy.

Cell therapy for tissue regeneration together with tissue engineering technology develops rapidly. Functional cell sheets for transplants are formed by cells. Biomaterials serve as cell carrier. The cell sheets can be easily detached and maintain the complete connective matrix in cells, cell phenotype, proliferation and differentiation. In our study, periosteum-like cell sheets were formed for orthopedic tissue regeneration. The PPCs and chondrocytes were used to produce periosteum-like cell sheets. The PPC carriers were prepared by polymerization of fibrinogen monomers mixed with thrombin. Natural acellular SIS was used as delivery vehicle for the cell sheets. The PPC sheets were placed onto a SIS layer thereby forming an artificial periosteal bilayer. The PPC sheets were wrapped around the tendon graft and placed into the bone tunnel. Histology showed higher matrix deposition with fibrocartilage formation at the tendon-bone junction after four weeks. Our results suggest that well-organized and functional PPC sheets maintain their differentiated capacity and keep their ex vivo osteochondral potential. The PPC sheets could act as periosteum to offer a novel approach to enhance the healing at the tendon-bone junction.

## Conclusion

Successful ACL reconstruction with a tendon graft requires solid healing of the tendon graft in the bone tunnel. Recent improvements in ACL reconstruction techniques have highlighted the importance of new biologic strategies to promote the intraarticular and intraosseous healing. Future techniques to improve the tendon-to-bone healing may include the use of cytokines, which will provide important signals for tissue formation and differentiation, gene therapy techniques, which will ensure the prolonged presence of molecules important for the healing process, stem cells, which will help produce a population of undifferentiated cells, and transcription factors, which will help direct nuclear gene expression. In addition, platelet-rich plasma or platelet gel from autologous blood facilitates delivery of serum-derived cytokines. Several techniques are being developed to inhibit the expression of molecules that might prohibit successful healing. Furthermore, modulation of the biomechanical environment may have profound effects on the cellular and molecular events at the healing tendon-bone interface [62]. These biologic strategies are currently under investigation, and will be applied in the clinical setting in the near future.

## Competing interests

The author declares that they have no competing interests.

## Authors' contributions

CHC prepared all the manuscript.
